# A comparison study of five different methods to measure carotenoids in biofortified yellow cassava (*Manihot esculenta*)

**DOI:** 10.1371/journal.pone.0209702

**Published:** 2018-12-28

**Authors:** Angélica M. Jaramillo, Luis Fernando Londoño, Juan Camilo Orozco, Gelver Patiño, John Belalcazar, Fabrice Davrieux, Elise F. Talsma

**Affiliations:** 1 HarvestPlus, International Center for Tropical Agriculture (CIAT), Cali, Valle del Cauca, Colombia; 2 Cassava program, International Center for Tropical Agriculture (CIAT), Cali, Valle del Cauca, Colombia; 3 UMR Qualisud, Agricultural Research for Development (CIRAD), Saint-Pierre, La Réunion, France; 4 Division of Human Nutrition and Health, Wageningen University, Wageningen, Gelderland, The Netherlands; Huazhong University of Science and Technology, CHINA

## Abstract

The most commonly used method for measuring carotenoid concentration is high-performance liquid chromatography (HPLC). Nevertheless, easier, quicker, and less costly proxy methods exist. We aimed to determine the diagnostic performance of several proxy methods: the spectrophotometer, iCheck Carotene, and near-infrared spectroscopy using both a desktop (dNIRS) and a portable (pNIRS) device for the measurement of total carotenoid concentration (TCC) and *all-trans-*β-carotene concentration (trans-BC) in 30 fresh cassava (*Manihot esculenta* Crantz) storage roots in comparison with HPLC. The spectrophotometer presented the highest predictability for TCC, followed by iCheck, dNIRS, and pNIRS. The dNIRS showed the highest predictability and agreement for trans-BC. The pNIRS showed the poorest repeatability and greatest underestimations compared with HPLC. The agreement between all methods was lower for higher carotenoid concentration, with the exception of the spectrophotometer. According to our results, and for screening purposes, the measurement of carotenoids in fresh cassava roots can be carried out by spectrophotometer, iCheck Carotene and NIRS methods depending on the availability of equipment.

## Introduction

Biofortification is the process by which the nutritional quality of staple crops is improved through of plant breeding, modern biotechnological techniques and/or agronomic practices. It provides an inexpensive, cost-effective, sustainable, and long-term complementary strategy to deliver more micronutrients by the consumption of foods that are already regularly consumed.[1]

Biofortified yellow cassava with increased carotenoid content has been developed through conventional plant breeding techniques at the International Center for Tropical Agriculture (CIAT).[2] Several studies have reported *all-trans-*β-carotene as the predominant carotenoid in yellow cassava storage roots. In the human body, carotenoids are converted into vitamin A and therefore this yellow cassava has great potential to alleviate vitamin A deficiency in cassava consuming populations. [3–6] In a plant breeding program for yellow cassava, thousands of roots need to be quickly screened and quantified for carotenoid concentration to enable breeders to make their selection based on carotenoid concentration.[4] Consequently, there is a need for the use of quick, reliable, and low-cost validated methods to measure carotenoid concentration in biofortified cassava.

The gold standard method for measuring carotenoid concentration is high-performance liquid chromatography (HPLC), which has the ability to separate and quantify individual carotenoids differing in their provitamin A activity.[7] However, HPLC analysis is costly and time-consuming, and requires a sophisticated laboratory with rigorous quality control and substantial technical resources.[8] Easier, quicker, and less costly proxy methods exist such as the spectrophotometer, iCheck Carotene, and near-infrared spectroscopy (NIRS) using desktop (dNIRS) and portable (pNIRS) devices. The spectrophotometer for carotenoid measurements is widely and routinely used in crops in which *all-trans-*β-carotene is the predominant carotenoid,[4,8–11] it also has been the standard method for the AOAC.[12] iCheck Carotene (BioAnalyt) is a novel portable spectrophotometric method to measure total carotenoids equipped with disposable extraction vials for ease of use. Other devices produced by BioAnalyt for the evaluation of different analytes have produced satisfactory results [13–15], but the use of iCheck Carotene in cassava roots is less well documented.[16] NIRS technology (both desktop and portable) has been proven to be an efficient and low-cost method when large numbers of samples need to be analyzed, but it requires a long-calibration procedure based on the laboratory reference method.[4,17–19] Carotenoid measurements with portable NIRS devices have been documented widely for other crops [20,21], but few trials have been carried out in cassava.[22] The accuracy of these methods to measure carotenoids and ease of use in comparison with HPLC have not been yet tested for yellow cassava.

During the processing of yellow cassava, carotenoids are degraded to a certain extent depending on the time and method of processing and type of variety used.[6,23,24] That is why carotenoid retention studies are critical to understand the effect of processing on carotenoid stability, allowing the identification and selection of those cassava cultivars with higher retention properties.[25,26] Retention measurements using portable devices would enable a breeding program to screen a number of genotypes to determine carotenoid losses in settings with no access to sophisticated equipment.

In this article, we aim to assess the diagnostic performance of the spectrophotometer, iCheck Carotene, dNIRS, and pNIRS in comparison with HPLC for measuring total carotenoid concentration (TCC) and/or *all-trans-*β-carotene concentration (trans-BC) in fresh cassava roots. Furthermore, we tested an improvement of the protocol for carotenoids extraction in fresh cassava roots for iCheck Carotene, as well as the feasibility of this method to measure the retention of total carotenoids in boiled biofortified cassava in comparison with HPLC.

## Materials and methods

Three different studies were conducted. The first study, called the “method comparison study”, was done to determine the diagnostic performance of the spectrophotometer, the iCheck Carotene, the dNIRS and the pNIRS in measuring TCC and trans-BC in fresh cassava roots in comparison with HPLC as the gold standard method. Despite that spectrophotometer, iCheck Carotene, dNIRS, pNIRS and HPLC are the names of laboratory devices, we used these names to refer to the extraction or quantification method for each of them. We also compared these methods based on analysis time per sample, number of samples per day, analysis cost per sample and the cost of equipment. To represent those differences, we set the HPLC values as 100%, and the others methods were compared with these. Analysis time per sample includes the time of extraction and running time of the equipment. Number of possible samples per day refers to the number of samples processed by one person. Analysis cost per sample includes the cost of the chemist, consumables, maintenance and depreciation of the equipment. Cost of equipment refers to the initial investment in the equipment. All prices do not include laboratory facilities nor institutional charges. The second study, named the “iCheck Carotene adaptation study”, evaluated a modification in the extraction protocol of the iCheck Carotene method. The third study, called the “retention comparison study”, assessed whether the iCheck Carotene method could be used to measure the degradation of carotenoids in yellow cassava after boiling. All extractions and carotenoid measurements were performed within 6 hours after harvesting and samples were prepared in duplicate under yellow light to avoid carotenoid isomerization.

### Cassava samples

Biofortified cassava roots were grown for 11 months at the CIAT Experimental Station in Palmira, Valle del Cauca Department, Colombia. All genotypes used are from the cassava breeding program of CIAT and were obtained from a rapid cycling recurrent selection.[10] The roots were harvested in the morning and prepared for analysis the same day.

### Method comparison study

Thirty yellow cassava genotypes were selected based on a wide variety range of TCC (1–31 μg/g). Three to five roots were washed, peeled, and ground in a food processor (Essen Skymsen Model PA-7SE). Mashed samples were packed in plastic bags, stored in a cooler box with ice bags, and protected from direct sunlight during transportation to the laboratory for further processing and analysis.

### iCheck Carotene adaptation study

Ten yellow cassava genotypes with a range of 8 to 25 μg/g of TCC were selected from previous dNIRS data. Roots were harvested and transferred to the laboratory where samples were cleaned, peeled, and cut in lengthwise quarters. Subsequently, two opposite quarters were selected and cut in cubes (~0.5 cm) with a stainless-steel knife. From these cubes, 50 g were ground in a homogenizer Grindomix GM200 (Retsch GmbH, Haan, Germany) and analyzed by HPLC and iCheck Carotene.

### Retention comparison study

Five additional genotypes with high carotene content (>14 μg/g TCC), selected from previous dNIRS data, were analyzed fresh and after boiling by iCheck Carotene and HPLC. Roots were cleaned and peeled, root ends were discarded. A disk of 6-cm-length and 5–6 cm of perimeter was selected and cut in half lengthwise, remaining parts were cut in ~0.5-cm cubes. The 6-cm cassava portions were boiled in a 1-L beaker with 800 mL of deionized water until fork-tender (from 25 to 40 min) and removed from the beaker to cool. Both fresh and boiled material were analyzed within the hour by HPLC and iCheck Carotene.

### Spectrophotometer and HPLC

The spectrophotometer was used to measure TCC and the HPLC was used to measure TCC and trans-BC. Carotenoids in cassava samples were extracted following the protocol described in the literature with modifications.[5] Five g of homogenized cassava sample were added to 50-mL centrifuge tubes with 10 mL of acetone. After 10 min, 10 mL of petroleum ether were added and mixed using an ultra-turrax (IKA Janke & Kunkel) for 30 seconds. The samples were then centrifuged (Eppendorf 5804R, Hamburg, Germany) at 3000 rpm for 10 min at 10 °C. The upper (organic) phase was collected separately and the extraction was repeated two additional times with 5 mL of acetone and 5 mL of petroleum ether. Ten mL of NaCl 0.1 M solution were added to the organic extract and centrifuged at 3000 rpm for 7 min at 10 °C. The lower (aqueous) phase was discarded and the washing process was repeated twice. Because of the wide range of carotenoid concentration, final extraction volumes of 30, 35, and 40 mL were adjusted for low, middle, and high TCC samples. An in-house quality control sample of freeze-dried orange fleshed sweet potato was stored at -80 °C and used to assess between-run and within-run variations. From the obtained extracts, TCC was measured in the μQuant spectrophotometer (BioTek Instruments, Inc., Winooski, VT) at an absorbance of 450 nm using an absorption coefficient (A^1%^_1cm_) of 2592 for *all-trans-*β-carotene in petroleum ether.[9,27]

For HPLC determinations, the organic extract measured in the spectrophotometer was totally dried in glass tubes with the nitrogen evaporator N-EVAP 112 (Organomation Associates, Berlin, MA) and re-dissolved in 3, 4, or 5 mL of (1:1) methanol and methyl tert-butyl ether for low, middle, and high TCC samples, respectively. The samples were shaken in a vortex mixer and filtered through a 0.22-μm polytetrafluoroethylene filter.

The chromatographic system consisted of a YMC Carotenoid S-5 C30 reversed-phase column (4.6 mm × 150 mm; particle size, 5 μm) with a YMC Carotenoid S-5 guard column (4.0 mm × 23 mm). Mobile phase A was methanol with 2% of ammonium acetate adjusted to pH 4.6 and mobile phase B was methyl tert-butyl ether. The gradient was 0 min 85% A, 20.0–23.5 min 40% A, and 23.8 min 85% A at a constant flow of 0.66 mL min^-1^. The column temperature was 25 °C, the autosampler temperature was 4 °C, and the injection volume was 10 μL. *All-trans-*β-carotene was identified and quantified with the use of an external standard and a calibration curve made in the range of 2–30 μg/mL (Sigma-Aldrich) at 450 nm using a diode array detector. TCC by HPLC was calculated using the sum of the areas of all peaks in the chromatogram at 450 nm and quantified with the same curve as trans-BC.[4]

### iCheck Carotene

The iCheck method was used to measure TCC only. The portable device consists of a hand-held photometer iCheck Carotene (BioAnalyt GmbH, Teltow, Germany) and the disposable reagent vials in which the extraction was performed. Five grams of homogenized sample were pounded into a mortar, 5 mL of distilled water were added, and grinding was performed until a smooth paste was obtained. The mashed root paste was transferred into a 50-mL centrifuge tube and the mortar and pestle were washed with 10 mL of distilled water, followed by its pouring into the centrifuge tube. The volume was adjusted to 25 mL with distilled water and the tube was shaken thoroughly until a homogeneous slurry was obtained. A total of 0.4 mL of the slurry was taken up with a syringe and injected into the reagent vial. After shaking the vial vigorously for 10 seconds, the sample was left to rest for 5 min and measured in the iCheck Carotene device.

### Desktop NIRS

NIRS methods were used to measure TCC and trans-BC. The dNIRS sample capsules were filled in duplicate with approximately 8 g of homogenized sample and analyzed using a FOSS 6500 monochromator with autocup sampling module (FOSS, Hillerod, Denmark). Samples were scanned in diffuse reflectance between 400 nm and 2500 nm with a 2-nm step and saved as the average of 32 scans. Instrument control was performed with the ISIscan Routine Analysis Software (Infrasoft International LLC, State College, PA).

The prediction model used for dNIRS was made with data generated during an eight-year period (2009 to 2016) using a local regression algorithm to predict values of TCC and trans-BC.[28] A total of 4606 samples in a range of 0.11 to 29.02 μg/g for TCC and 0.00 to 20.54 μg/g for trans-BC were used for the calibration and validation of the prediction model.

### Portable NIRS

Right after carotenoid measurements in the dNIRS, the same dNIRS sample capsules were scanned in a portable LabSpec4 Standard-Res spectrometer (Analytical Spectral Devices-ASD Inc., Boulder, CO) equipped with an ASD fiber-optic high-intensity contact probe. Spectra were recorded in a range of 350 to 2500 nm at 1-nm intervals and saved as a 50-scan average. Instrument control was performed with the Indico Pro software package (Analytical Spectral Devices-ASD Inc., Boulder, CO).

The pNIRS prediction model was used during the cassava breeding season in 2015 with 699 samples of fresh cassava. Among the 699 samples, 183 were analyzed in the laboratory with values of 10.37 to 28.54 μg/g for TCC and 7.49 to 20.54 μg/g for trans-BC. The remaining 516 samples were analyzed using the dNIRS calibrations developed at CIAT [28] with values of 4.57 to 24.57 μg/g for TCC and 1.97 to 15.46 μg/g for trans-BC. The results for all 699 samples (laboratory and dNIRS predictions) were used to calibrate the pNIRS based on MPLS regression method. The spectra from pNIRS were trimmed in order to develop calibration with Win-ISI 4.0 software (Infrasoft International and FOSS, Hillerod, Denmark).

### Dry matter content

Three grams of the ground root tissue were weighed and dried in an oven at 105 °C. After 24 hours, the samples were cooled in a desiccator and weighed. Dry matter was expressed as the percentage of dry weight relative to fresh weight. Dry matter measurements were performed in triplicate.

### Data analysis

Data processing was done and descriptive statistics were generated using Microsoft Office Excel 2013 and R studio (v3.4.2). Differences between HPLC and proxy methods were normally distributed except for trans-BC dNIRS. For trans-BC dNIRS, two outliers were identified by box-plot graphs and the exclusion resulted in normally distributed data without affecting our conclusions. For pNIRS, five genotypes were excluded for quantification because they were out of the calibration range of the curve (<2.0 for trans-BC or <4.6 for TCC and >20.5 μg/g for trans-BC or >28.5 μg/g for TCC). Also for the pNIRS method, one sample predicted a negative value for TCC, and a value of zero was assigned.

Scatter plots and regression analysis were used to assess associations between the different methods and HPLC. Bland Altman plot analysis was used to assess the agreement between the different methods and the HPLC method. The Bland Altman plot analysis is a simple illustrative way to test the interchangeability of methods by plotting the mean of the methods on the X-axis and the difference of the methods on the Y-axis. Limits of agreement are defined as the mean difference ± 1.96 standard deviations of the differences.[13,15,29,30]

The between-run coefficients of variation (CV) obtained with the in-house quality control sample were 2.8%, 1.7%, and 4.0% for TCC in HPLC, the spectrophotometer, and iCheck Carotene, respectively, and 2.9% for trans-BC in HPLC. Within-run CVs for HPLC were 0.4% and 0.3% for TCC and trans-BC, respectively.

Cooking retention was calculated using the apparent retention equation (Eq 1).[9,31]

$$\%\text{Retention}=\frac{\text{Carotenoid}\;\text{content}\;\text{per}\;\text{g}\;\text{of}\;\text{cooked}\;\text{food}\;(\text{dry}\;\text{basis})}{\text{Carotenoid}\;\text{content}\;\text{per}\;\text{g}\;\text{of}\;\text{fresh}\;\text{food}\;(\text{dry}\;\text{basis})}\times 100$$ (1)

## Results and discussion

### Method comparison study

**Fig 1:**
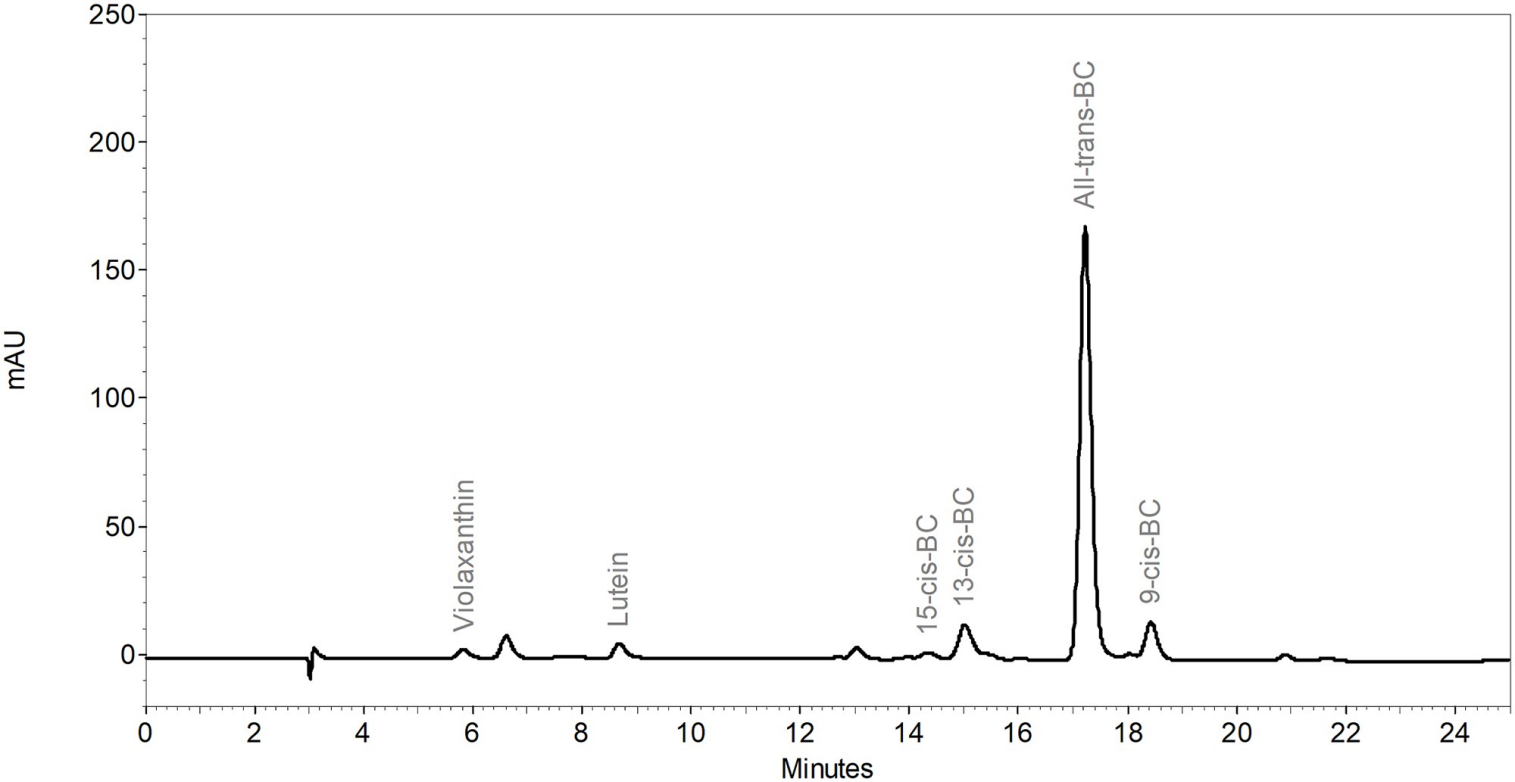
HPLC chromatogram for cassava storage roots (detection at 450 nm). HPLC conditions are described in the text.

*All-trans-*β-carotene was the predominant carotenoid in the cassava storage root samples (Fig 1), ranging from 35% to 84%. The mean of the TCC for the cassava samples analyzed by HPLC was 15.0 μg/g, with a range of 1.0 to 30.7 μg/g (Table 1). The dNIRS method showed the lowest differences between the means in TCC and trans-BC in comparison with HPLC (0.2 μg/g and 0.4 μg/g, respectively), followed by the spectrophotometer with a difference of 0.7 μg/g in TCC. Results with iCheck Carotene were on average 1.6 μg/g lower than the TCC HPLC and the highest differences were found in pNIRS for TCC (-2.8 μg/g) and trans-BC (-0.8 μg/g) with underestimations of 19% and 8%, respectively. pNIRS also presented the lowest repeatability between duplicate measurements with CVs of 12.1% and 7.8% for TCC and trans-BC, respectively. However, the highest CV values in pNIRS are mostly in low TCC and trans-BC samples (<4 μg/g), greatly affecting the mean CV of the method. HPLC, the spectrophotometer, iCheck Carotene, and dNIRS exhibited CV values between 1.7% and 3.4%, all acceptable values for repeatability.[32]

**Table 1:** TCC and trans-BC in fresh cassava samples measured by HPLC, spectrophotometer, iCheck Carotene, dNIRS, and pNIRS.

	TCC HPLC	TCC Spec.	TCC iCheck	TCC dNIRS	TCC pNIRS	trans-BC HPLC	trans-BC dNIRS	trans-BC pNIRS
**Number of samples**	30	30	30	30	25	30	28	25
**Min (μg/g)**	1.0	1.1	1.8	0.9	0.0	0.4	0.3	0.4
**Max (μg/g)**	30.7	30.0	24.7	25.4	20.3	20.0	17.9	15.7
**Mean (μg/g)**	15.0	14.3	13.4	15.2	12.2	10.1	9.7	9.3
**CV duplicate samples (%)**	2.4	2.3	3.4	1.7	12.1	3.2	2.4	7.8

Values reported in fresh weight.

**Fig 2:**
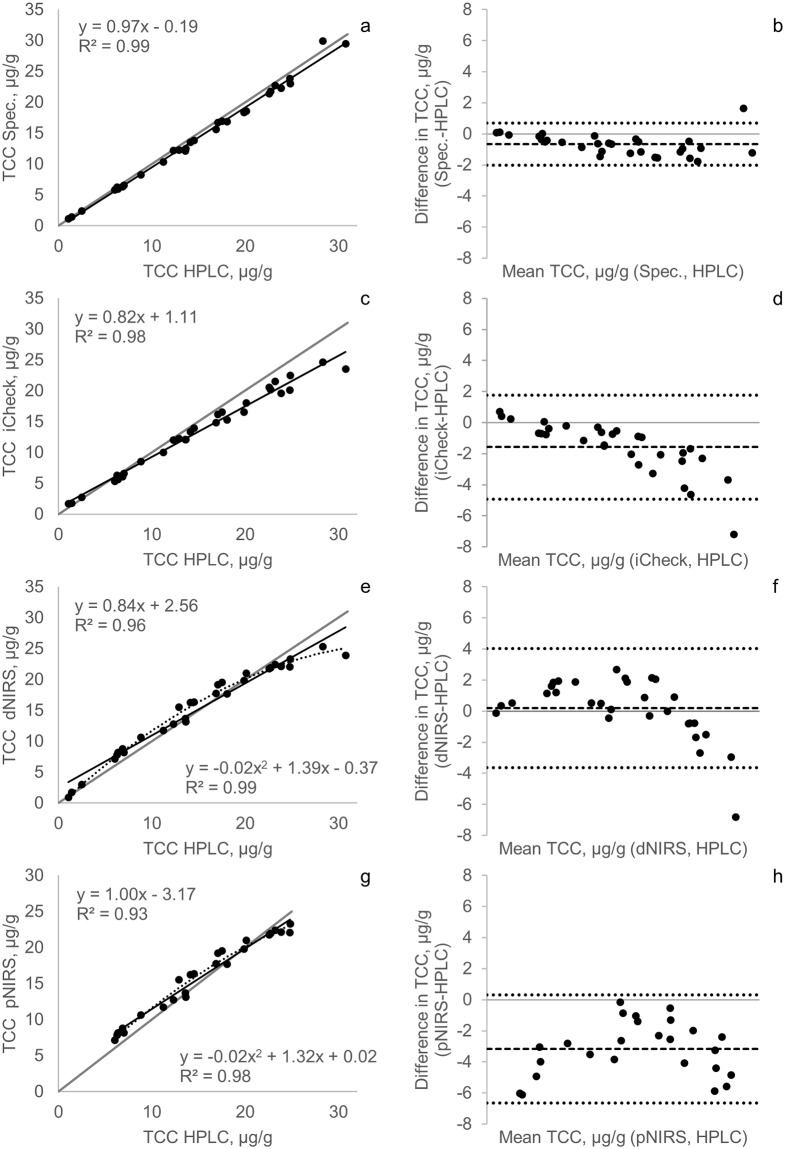
Scatterplots and Bland Altman comparison between TCC measured by HPLC versus spectrophotometer (a-b), iCheck Carotene (c-d), dNIRS (e-f), and pNIRS (g-h). The black lines indicate linear regression lines and the dotted lines indicate quadratic regression lines. Gray lines represent the line of identity. The square dotted lines represent the bias and the round dotted lines indicate the 95% confidence interval.

**Fig 3:**
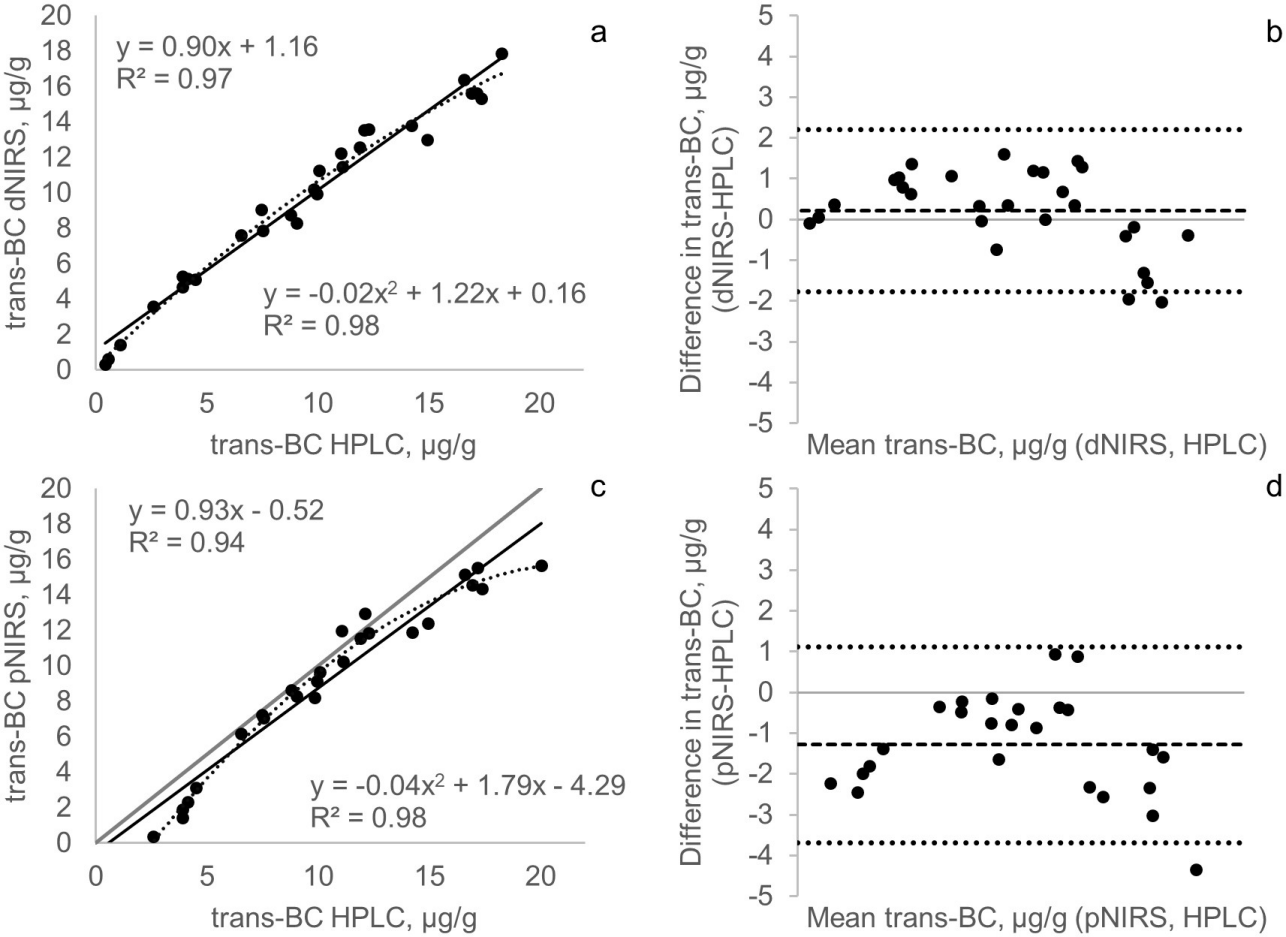
Scatterplot and Bland Altman comparison between trans-BC measured by HPLC versus dNIRS (a-b) and pNIRS (c-d). The black lines indicate linear regression lines and the dotted lines indicate quadratic regression lines. Gray lines represent the line of identity. The square dotted lines represent the bias and the round dotted lines indicate the 95% confidence interval.

The spectrophotometer method presented the highest coefficient of determination with HPLC (*r*^2^ = 0.99, *p* < 0.001) for TCC, followed by iCheck Carotene (*r*^2^ = 0.98, *p* < 0.001) (Fig 2a and 2c). Nevertheless, only the spectrophotometer regression line fitted with the identity line according to the regression analysis (95% confidence intervals), which means there is no significant difference between methods. For dNIRS and pNIRS, the coefficients of determination were also high (*r*^2^>0.93, *p* < 0.001) for both TCC and trans-BC, and improved when quadratic regressions were used for both TCC (*r*^2^>0.98) (Fig 2e and 2g) and trans-BC (*r*^2^>0.98) (Fig 3a and 3c). Figs 2e, 2g, 3a and 3c show the improvement in the correlation coefficients from linear to quadratic regressions for dNIRS and pNIRS methods (for TCC and trans-BC). Non-linear fitting for higher values of TCC and trans-BC in fresh cassava samples was previously observed.[17]

Bland Altman plots showed that HPLC had the best agreement with the spectrophotometer with a bias of -0.7 μg/g and limits of agreement of -2.0 and 0.7 μg/g (Fig 2b). In contrast, a previous study showed an overestimation for the spectrophotometer when comparing carotenoid concentration with HPLC, probably because of the use of the *all-trans-*β-carotene absorption coefficient to quantify total carotenoids in spectrophotometric analysis in samples with high amounts of carotenoids different from *all-trans*-β-carotene.[13]

The iCheck Carotene method showed a bias of -1.6 μg/g and limits of agreement of -4.9 and 1.8 μg/g in the Bland Altman plot (Fig 2d). Also, an underestimation for most of the samples was observed and this increased with higher carotenoid concentration (>15 μg/g). These differences between iCheck Carotene and HPLC were not found when *all-trans*-β-carotene was measured in whole blood and plasma [29] or egg yolks [13], suggesting that the iCheck behavior is matrix-specific.

dNIRS and pNIRS methods (for TCC and trans-BC) presented a quadratic behavior in the Bland Altman plots as was previously shown with linear regression (Figs 2f, 2h, 3b and 3d). The dNIRS method produced good agreement with HPLC but the differences are bigger with higher TCC and trans-BC concentrations (>23 μg/g for TCC and >18 μg/g for trans-BC), making predictions less precise at higher carotenoid concentrations. We were not able to conduct pNIRS analysis in all samples because the calibration range was shorter than the range of the analyzed samples. The Bland Altman plot (Figs 2h and 3d) showed pNIRS underestimation for most of the samples analyzed, resulting in a bias of -3.2 and -1.28 μg/g for TCC and trans-BC, respectively. Prediction in NIRS technology depends entirely on the reliability of primary calibration data [18], and therefore it is necessary to continue including high carotenoid samples in the dNIRS calibration to improve predictions in this part of the curve and to develop new evaluations to measure whether improvement was achieved. For pNIRS, it is necessary to develop a new large calibration set that includes samples from all ranges of possible concentrations and their corresponding laboratory data.

**Fig 4:**
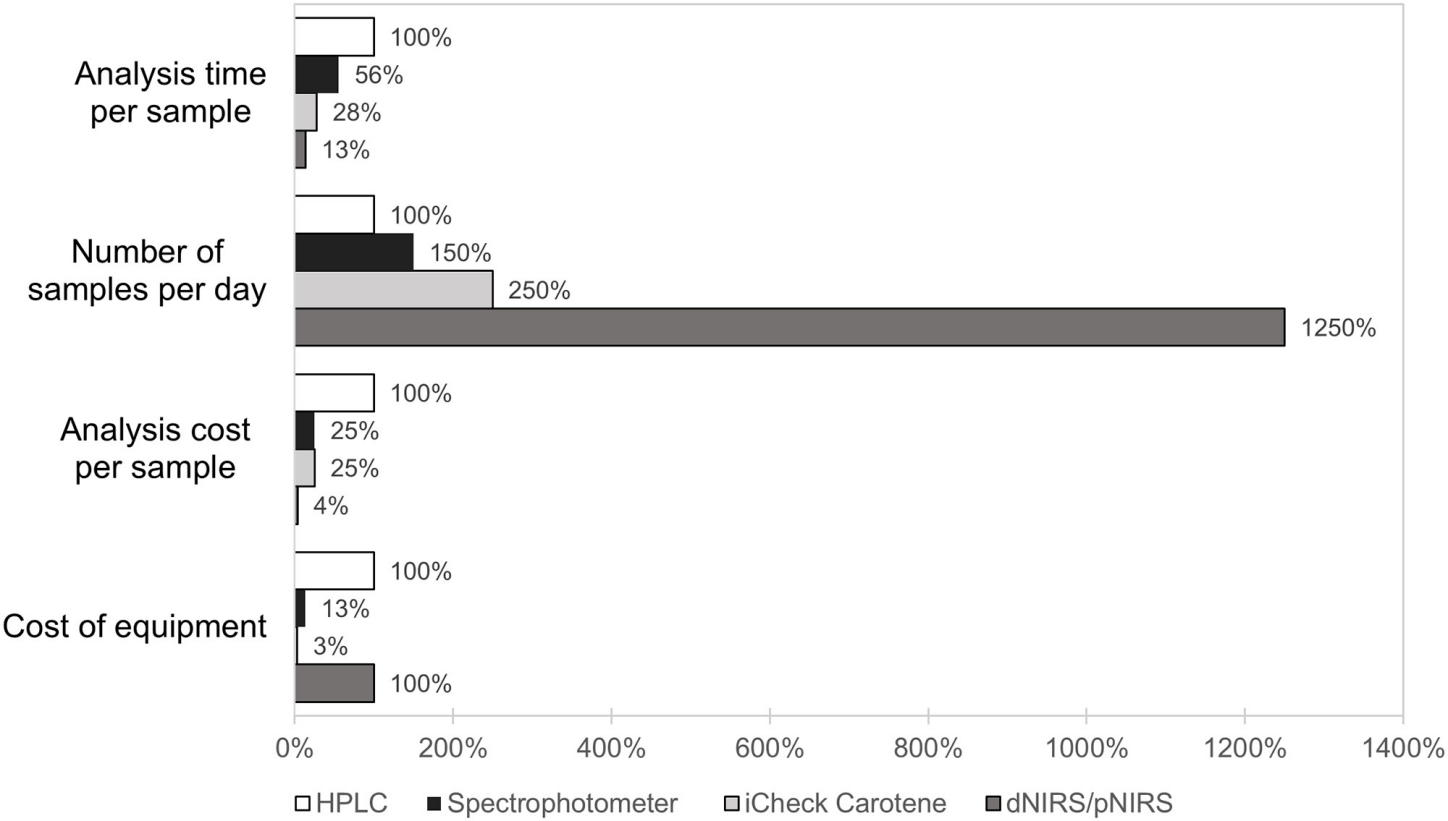
Time and price comparison for carotenoid methods.

The spectrophotometer, iCheck Carotene, dNIRS, and pNIRS are all proxy methods that are easier, quicker, and less costly than HPLC. However, they vary in usefulness, strengths, and limitations (Fig 4). The spectrophotometer and iCheck Carotene are not able to measure individual carotenoids, although HPLC, dNIRS, and pNIRS could discriminate between TCC and trans-BC values. Laboratory facilities are necessary to conduct extractions for HPLC and the spectrophotometer, the dNIRS instrument has to be in controlled conditions for humidity and temperature. pNIRS can be used in remote areas without access to laboratory infrastructure, although a grinder is needed to homogenize the sample before analysis. The iCheck Carotene is a method that can be used without laboratory facilities or even without electricity, which makes it useful in remote areas. dNIRS and pNIRS methods are convenient when routine analysis of thousands of samples is needed due to their ability to analyze more than 100 samples daily. The accurateness of the dNIRS or pNIRS depends on the calibration with HPLC, which is the main limitation of this method as a sufficient number of samples covering the range of variability of the constituent is needed.[33]

HPLC and the spectrophotometer are both more time-consuming methods than the others presented because of long extraction steps and sample run time in the case of HPLC. The iCheck Carotene requires a manual extraction process, for which time and labor will vary depending on the sample hardness. In the case of NIRS methods, both sample processing and measurement are fast and relatively simple. The number of samples per day that can be analyzed with a certain method is directly related to the analysis time required per sample.

HPLC is the most expensive method due to its labor intensiveness required for analysis. Also, the high-purity reagents and the use of standards, filters, and nitrogen are expensive and affect the total cost. The spectrophotometer method also requires a substantial amount of reagents and long staff labor time. By contrast, the equipment reading is easy and fast, which reduces the costs compared with HPLC. The iCheck Carotene cost per sample depends mostly on the disposable vials cost. In the case of NIRS methods, the cost per sample is very low and it is mostly associated with the cost of the staff.

HPLC, dNIRS, and pNIRS present a similar equipment cost but it must be noticed that for the development of the NIRS methods, an HPLC method is required to create and maintain the calibration throughout the years. The spectrophotometer is a versatile equipment with a moderate cost and it is usually found in analytical laboratories. On the other hand, the iCheck Carotene requires the lowest initial investment but can be used only exclusively for carotenoid analysis.

### iCheck Carotene comparison study

**Fig 5:**
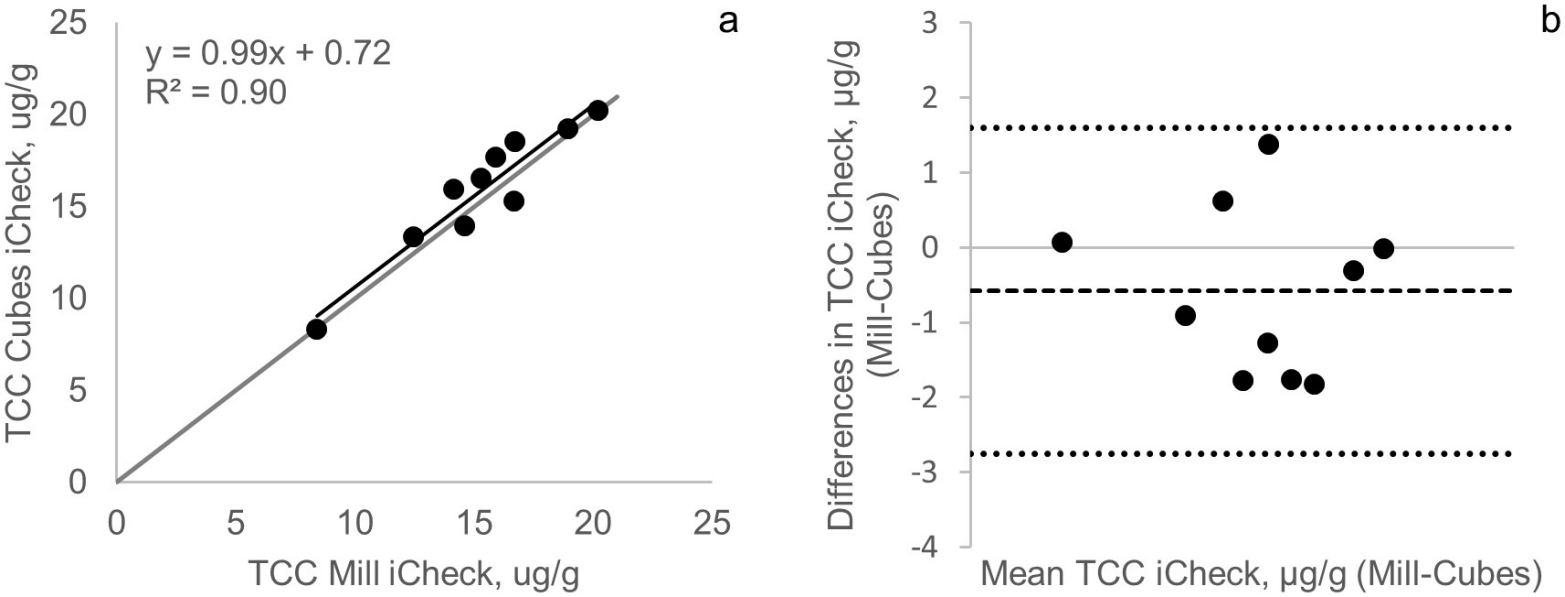
Scatterplot and Bland Altman comparison between total carotenoid concentration (TCC) in cubes and milled samples for iCheck Carotene. The black line indicates the linear regression line. The gray line indicates the line of identity. The square dotted line represents the bias and the round dotted lines represents the 95% confidence interval.

Comparison between cubes and ground fresh cassava samples showed a mean TCC concentration of 15.9 μg/g (2.9% CV) and 15.3 μg/g (2.0% CV), respectively. A high coefficient of determination (*r*^2^ = 0.90, *p* < 0.001) between the two methods was observed, and both the confidence intervals (95%) of the slope as well as the intercept fell between the identity line, suggesting that there is no significant difference between the methods. The Bland Altman plot also showed a good agreement between the methods with a bias of -0.6 μg/g and limits of agreement of -2.8 and 1.6 μg/g (Fig 5).

The results for cubes and ground samples are similar and, therefore, both methods of processing for extraction can be used interchangeably. The iCheck Carotene method was originally developed for cubes of ~0.5 cm cut manually by a knife, but mashing cubes with a mortar and pestle is time-consuming and labor-intensive when a large number of samples need to be analyzed. A modification of the method using a mechanical grinder reduced time and labor and resulted in similar values compared to using cubes. Samples must be manipulated minimizing oxidizing conditions to avoid carotenoid degradation.

### Retention comparison study

The mean concentrations and standard deviations for TCC and dry matter for fresh and boiled cassava roots are shown in Table 2. The mean of TCC concentration for HPLC and iCheck Carotene in boiled samples were 10.5 μg/g and 6.8 μg/g respectively, which indicates that the iCheck Carotene method generate lower values than HPLC in the cooked samples. Apparent retention measured by HPLC was between 54% and 79%, indicating a high variation between genotypes, what was also observed in a previous study with boiled cassava roots in which the variation was between 27% and 83% for six genotypes.[24] In contrast, iCheck Carotene showed an apparent retention between 31% and 58%, 20% lower than the HPLC outcome.

**Table 2:** TCC, dry matter, and apparent retention of five yellow cassava lines before and after boiling measured by HPLC and iCheck Carotene.

	Before boiling			After boiling			TCC apparent retention	
Clone	Dry matter (%)	TCC HPLC (μg/g)	TCC iCheck (μg/g)	Dry matter (%)	TCC HPLC (μg/g)	TCC iCheck (μg/g)	HPLC (%)	iCheck (%)
**GM 8351–1**	38.8 ± 0.7	17.8 ± 0.4	17.1 ± 0.8	37.4 ± 0.2	9.3 ± 0.2	5.1 ± 0.0	54	31
**GM 8409–13**	33.9 ± 1.0	17.6 ± 0.1	16.9 ± 0.3	32.2 ± 0.1	13.3 ± 1.3	8.6 ± 0.6	79	54
**GM 8413–1**	31.0 ± 0.3	16.5 ± 0.3	15.9 ± 0.3	31.0 ± 1.1	12.0 ± 1.6	9.2 ± 0.9	73	58
**SM 3882–9**	36.7 ± 0.6	18.2 ± 0.8	16.8 ± 0.2	34.9 ± 0.0	9.8 ± 0.7	5.9 ± 0.4	56	37
**SM 3882–76**	34.8 ± 0.1	14.4 ± 0.1	13.7 ± 0.5	35.4 ± 0.4	8.1 ± 1.1	5.3 ± 0.3	55	38
**Mean**	35.0	16.9	16.1	34.2	10.5	6.8	63	43

Values reported in fresh weight.

Values represent means ± SD of two replicates.

The large difference in apparent retention between HPLC and iCheck Carotene was caused by the lower values for TCC for boiled samples determined by iCheck Carotene. The starch concentration in the cassava root is more than 90%, measured in dry weight. During cassava boiling, the starch gelatinizes and hydrates,[34] which hinders carotenoids extraction due to their hydrophobic nature. The mechanical extraction with organic solvents might improve the extraction, which seems to be essential for proper carotenoid quantification when roots have been exposed to thermal processing. This is probably the reason for the low TCC values obtained for boiled samples analyzed by iCheck Carotene. More research is needed to assess the potential of iCheck Carotene for the evaluation of the carotenoid retention in samples subjected to thermal treatments.

## Conclusions

For screening purposes to measure carotenoid concentration in fresh cassava, the spectrophotometer as well as iCheck Carotene and dNIRS methods could be used according to equipment availability. However, iCheck Carotene and dNIRS should be used with caution when measuring cassava genotypes with carotenoid concentration higher than 15 μg/g for iCheck, higher than 23 μg/g for TCC and 18 μg/g for trans-BC in dNIRS, given that the agreement between these methods was lower for higher concentrations.

The pNIRS presented high CV values, an underestimation for carotenoid concentration in general, and a shorter range of concentrations. Therefore, we recommend developing a new calibration set with complete laboratory data to cover a wider range of concentrations and to have more data points to improve the agreement.

The protocol suggested by the iCheck Carotene manufacturer can be modified to a more user-friendly process without affecting the quality of the results.

Finally, the carotenoid retention in boiled cassava samples should not be measured with the iCheck Carotene method in its current form. It is necessary to conduct method-specific adjustments before a new validation study can be performed.
